# Stereotypes bias face perception via orbitofrontal–fusiform cortical interaction

**DOI:** 10.1093/scan/nsaa165

**Published:** 2020-12-03

**Authors:** Benjamin O Barnett, Jeffrey A Brooks, Jonathan B Freeman

**Affiliations:** Division of Psychology and Language Sciences, University College London, London WC1E 6BT, UK; Department of Psychology, New York University, New York, NY 10003, USA; Department of Psychology, New York University, New York, NY 10003, USA; Center for Neural Science, New York University, New York, NY 10003, USA

**Keywords:** social cognition, face perception, stereotypes, social vision, multivariate fMRI

## Abstract

Previous research has shown that social-conceptual associations, such as stereotypes, can influence the visual representation of faces and neural pattern responses in ventral temporal cortex (VTC) regions, such as the fusiform gyrus (FG). Current models suggest that this social-conceptual impact requires medial orbitofrontal cortex (mOFC) feedback signals during perception. Backward masking can disrupt such signals, as it is a technique known to reduce functional connectivity between VTC regions and regions outside VTC. During functional magnetic resonance imaging (fMRI), subjects passively viewed masked and unmasked faces, and following the scan, perceptual biases and stereotypical associations were assessed. Multi-voxel representations of faces across the VTC, and in the FG and mOFC, reflected stereotypically biased perceptions when faces were unmasked, but this effect was abolished when faces were masked. However, the VTC still retained the ability to process masked faces and was sensitive to their categorical distinctions. Functional connectivity analyses confirmed that masking disrupted mOFC–FG connectivity, which predicted a reduced impact of stereotypical associations in the FG. Taken together, our findings suggest that the biasing of face representations in line with stereotypical associations does not arise from intrinsic processing within the VTC and FG alone, but instead it depends in part on top-down feedback from the mOFC during perception.

We effortlessly extract social information when we encounter others’ faces, gaining insight into their identity, gender, race or emotion (Fiske and Neuberg, 1990; Ekman, 1993; Macrae and Bodenhausen, 2000). Such information enables us to better understand other people and, in the case of social categories, can often provide a lens for social interaction and a foundation for stereotyping and prejudice. The right fusiform gyrus (FG) plays a key role in faces’ social perception, helping represent a face’s identity, gender, race and emotion (Kanwisher and Yovel, 2006; Contreras *et al.*, 2013; Wegrzyn *et al.*, 2015) and showing a high sensitivity to faces’ social category cues (Freeman *et al.*, 2010; Stolier and Freeman, 2017).

Increasingly, social cognitive processes such as stereotypes, attitudes and goals have also been demonstrated to play a role in faces’ initial social perception (Adams *et al.*, 2011). For instance, numerous studies have demonstrated that the perception of faces may be influenced by stereotypes (Hugenberg and Bodenhausen, 2004; Johnson *et al.*, 2011; Freeman *et al.*, 2011b), motives and intergroup bias (Caruso *et al.*, 2009; Ratner *et al.*, 2014), social dominance orientation (Ho *et al.*, 2013), visual context (Freeman *et al.*, 2013, 2015) and political and economic factors (Krosch *et al.*, 2013; Krosch and Amodio, 2014), among others. Perceivers’ use of various forms of contextual information in perceiving faces’ emotion in particular has long been recognized (Russell, 1997; Barrett *et al.*, 2011).

Among the social cognitive processes that may influence perception, stereotypes and other kinds of social-conceptual knowledge may play a pronounced role. Stereotypes are learned semantic associations related to social categories, which are acquired from one’s social environment and not necessarily consciously endorsed (Macrae and Bodenhausen, 2000). A complex system of neural regions plays a role in multiple aspects of stereotyping, prejudice and intergroup biases (Knutson *et al.*, 2007; Mitchell *et al.*, 2009; Quadflieg *et al.*, 2009; Contreras *et al.*, 2012; Amodio, 2014; Hehman *et al.*, 2014; Freeman and Johnson, 2016; Mattan *et al.*, 2018; Bagnis *et al.*, 2019). In the context of initial social perceptions, current models such as the dynamic interactive (DI) model propose that, during the perception of another person, the medial orbitofrontal cortex (mOFC) accesses social-conceptual associations (including stereotypes) and provides top-down feedback to evolving face representations in right ventral temporal cortex (VTC) regions, particularly the right FG (Freeman and Ambady, 2011; Freeman and Johnson, 2016). Such mOFC top-down modulation would allow prior knowledge and social expectations to adaptively constrain face-related FG representations. Indeed, recent studies have shown that the representational structure of faces’ multi-voxel response patterns in the FG partly reflects expectations and social-conceptual knowledge, including stereotypes (Stolier and Freeman, 2016; Brooks *et al.*, 2019). More generally, studies have demonstrated the impact of a variety of social cognitive biases on FG activity (Van Bavel *et al.*, 2008; Ratner *et al.*, 2012; Brosch *et al.*, 2013; Kaul *et al.*, 2014; Bagnis *et al.*, 2020).

These social-visual interactions have been argued to draw on domain-general neural and computational mechanisms, similar to those involved in top-down modulation of object recognition. Consistent with this perspective, as with social perception, in object perception, multi-voxel response patterns in the VTC have also been found to reflect not only visual attributes but also abstract semantic relationships between object categories (Khaligh-Razavi and Kriegeskorte, 2014; Jozwik *et al.*, 2017; Storrs *et al.*, 2017). Several studies support the view that mOFC–FG interplay may drive social-conceptual impact on perception of faces. For instance, neuroimaging studies suggest that the mOFC provides perceptual priors for object-recognition processes in the VTC and FG (Bar, 2004; Bar *et al.*, 2006; Summerfield and Egner, 2009), and some object-recognition signals in the mOFC have been shown to temporally precede those in the VTC and FG using magnetoencephalography (Kveraga *et al.*, 2007). Moreover, expectations about faces in particular enhance top-down effective connectivity from the mOFC to the FG (Summerfield *et al.*, 2006; Summerfield and Egner, 2009).

An alternative possibility is that social-conceptual impact on the FG may arise gradually over extended periods of time via chronic mOFC-FG signaling, such that face-related FG representations come to conform to those signals on their own and no longer require top-down feedback during real-time perception (e.g. Khaligh-Razavi and Kriegeskorte, 2014; Jozwik *et al.*, 2017). As such, social-conceptual knowledge would come to manifest in the FG and other VTC regions in a more permanent manner. Critically, this alternative account diverges from the former in its prediction as to whether the impact of social-conceptual knowledge on face-related FG representations would persist even after functional connection with the mOFC has ceased.

One way to distinguish between these accounts and clarify the role of mOFC feedback in stereotypical impact on face perception is through backward masking. Backward masking involves a brief presentation of a target stimulus that is immediately replaced by a masking stimulus, which results in the target not being consciously reported by participants. Although masking renders visual stimuli subjectively invisible, VTC regions still exhibit extensive perceptual encoding of the stimuli (Dehaene and Naccache, 2001; Moutoussis and Zeki, 2002). However, the extent of reentrant feedback into VTC regions is extremely reduced during the processing of masked stimuli (Dehaene and Naccache, 2001; Dehaene and Changeux, 2011; Baars *et al.*, 2013). Given that frontal interactions with VTC are reduced under masked exposures, masking should reduce if not eliminate the social-conceptual impact on face-related FG representations—if indeed such impact depends on mOFC–FG interactions during perception. Previous work has shown that social-conceptual knowledge affects face perception and FG representational structure automatically and without explicit task demands (Stolier and Freeman, 2016; Freeman and Johnson, 2016; Brooks *et al.*, 2019). Here, we use backward masking to provide evidence that such automatic impacts due to stereotypes do not arise from VTC alone but require real-time input from outside VTC, namely the mOFC.

Numerous studies have demonstrated an impact on face perception due to social-conceptual processes, ranging from stereotypes to emotion concepts and person knowledge, among others (for review, Freeman *et al.*, 2020). In the present study, we make use of one well-studied impact of social-conceptual knowledge on face perception involving gender stereotypes. Men tend to be stereotyped as aggressive, and women tend to be stereotyped as docile; such stereotypical associations lead perceptions of male and female faces to be biased toward anger and happiness, respectively (Hess *et al.*, 2000, 2004; Brooks *et al.*, 2018). Moreover, individual differences in the strength of these stereotypic associations (male = angry and female = happy) predict the extent of an individual’s perceptual bias, i.e. male faces perceived angrier and female faces perceived happier (Brooks *et al.*, 2018). Neuroimaging studies have revealed that the representational structures of male and female faces in the FG and mOFC are biased in a consistent manner, whereby male and female faces evoke neural patterns more similar to the neural patterns associated with anger and happiness, respectively (Stolier and Freeman, 2016). Moreover, such effects correlate with individual differences in the strength of stereotypical associations. In the present study, we examined to what extent this stereotypical biasing in neural pattern similarity structure persists even when reentrant feedback to the VTC is relatively restricted via masking. Our aim is to provide evidence that mOFC–FG interplay is a mechanism by which stereotypes exert their influence on how faces are visually represented.

## Materials and methods

### Subjects

Forty adult, right-handed subjects were recruited from the New York City area (24 female; mean age = 23.38 years; 13 White, 7 Black, 9 Asian and 11 other). Subjects were financially compensated for participation. All subjects provided informed consent in a manner approved by the New York University Institutional Review Board and had no fMRI contraindications. One subject was found to be ineligible for the study after participating and was excluded. One subject was removed from analyses involving the stereotype content task for failing to follow instructions correctly in the task. Five subjects were excluded from fMRI analyses: two subjects because they demonstrated sensitivity to the masked stimuli in their conscious responses (see ‘Results’ section), two subjects based on excessive movement and one subject for failing to comply with instructions (failed to make button responses). This resulted in a final fMRI sample of 34 subjects (22 female; mean age = 23.50 years; 12 White, 7 Black, 7 Asian and 8 other).

### Stimuli

**Fig. 1.**
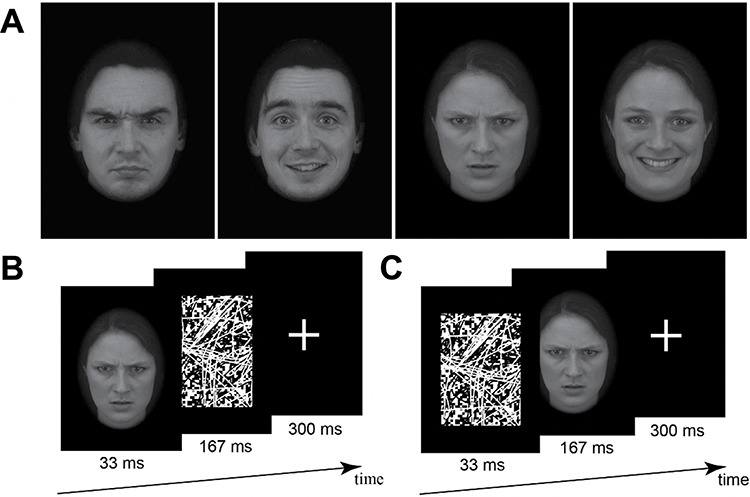
(A) Example face stimuli. Examples of angry male, happy male, angry female and happy female face stimuli used in the fMRI, discrimination and mouse-tracking tasks. fMRI procedure. (B) Masked condition and (C) unmasked condition. In both, each sequence was repeated four times within a trial (totaling 2000 ms).

Face stimuli consisted of 140 faces depicting 70 male individuals and 70 female individuals (all White) each displaying angry and happy expressions. This made for four stimulus conditions: sex (male *vs* female) × emotion (angry *vs* happy). These 140 images comprised the full set of directly oriented face stimuli for the angry and happy categories from the well-validated Karolinska Directed Emotional Faces database (Lundqvist *et al.*, 1998). Using normed ratings from previous validation studies (Garrido and Prada, 2017), emotional intensity did not differ between angry *vs* happy expressions of the male faces (*t*_(34)_ = 1.16, *P* = 0.253) or angry *vs* happy expressions of the female faces (*t*_(34)_ = 0.19, *P* = 0.852); nor did emotional intensity differ between the angry male *vs* angry female targets (*t*_(33)_ < 0.01, *P* = 0.997) or happy male *vs* happy female targets (*t*_(33)_ = 1.17, *P* = 0.250). All stimuli were edited with a black vignette such that only the face was visible against a black background (Figure 1A). The stimuli were additionally matched on luminance and contrast across the four stimulus conditions using the SHINE toolbox (Willenbockel *et al.*, 2010). We used black and white visual noise patterns as backward masks.

### Procedure

#### fMRI task.

We employed a backward masking paradigm adapted for rapid event-related fMRI using a similar procedure as a previous study (Freeman *et al.*, 2014). Subjects were presented with four runs (two masked and two unmasked). Masked runs always preceded unmasked runs, as unmasked runs presented first could increase subjects’ sensitivity to masked targets before their masked presentation. Within masked runs, each trial consisted of a face stimulus (33 ms), followed by a pattern mask (167 ms) and a fixation cross (300 ms), which was repeated four times and totaling 2000 ms (1 TR), see Figure 1B. Each 2000 ms presentation was treated as one trial. In unmasked runs, the pattern mask and face stimulus were presented in reversed order (33 ms visual pattern + 167 ms face stimulus + 300 ms fixation cross), thereby ensuring identical visual information across masked and unmasked conditions (Figure 1C). Within each run, half of the face stimuli were presented twice along with 77 null events (2000 ms fixation cross). The particular stimulus ordering in each condition was counterbalanced across subjects. All events within runs were sequenced in a manner to optimize the efficiency of event-related BOLD signal estimation using *optseq2* (Dale, 1999). In order to maintain subjects’ attention, subjects were asked to report via button press whenever the fixation cross was blue (25% of trials). Subjects were not asked to attend to the gender or emotion of the faces during this task.

#### Masked discrimination task.

To provide an objective measure of sensitivity to the face stimuli during masked presentations, we used a visual discrimination task. Following the fMRI task, while still in the scanner, subjects were presented with each of the faces used in the fMRI task. They were told in advance that they would be presented with masked faces and instructed to categorize their gender as accurately as possible. The stimuli were presented in the identical procedure to how they were presented in the masked condition of the fMRI task, except here trials were self-paced and subjects were prompted for a categorization (male or female?) at the end of each 2000 ms presentation. Each face stimulus was presented once in this task, totaling 140 trials.

#### Mouse-tracking categorization task.

To measure stereotypical biases during perception, we used computer mouse-tracking. Mouse-tracking paradigms measure the extent of social category co-activation during categorization tasks. During two-choice categorization tasks (e.g. angry *vs* happy), deviation in a subject’s hand trajectory toward each category response provides an indirect measure of the degree to which that category was activated during perception. If stereotypical associations link one category to another (e.g. male to anger), subjects’ perceptions are biased toward that category and, consequently, their hand trajectories deviate toward that category response in mouse-tracking tasks. Mouse-tracking is a well-validated methodology and has long been used to provide evidence for social-conceptual impact on face perception, including stereotypes (Freeman *et al.*, 2011a; Freeman, 2018).

Following the post-scan discrimination task, participants completed a standard two-choice categorization task, implemented in MouseTracker software (Freeman and Ambady, 2010). The face stimuli used were the same as those from the fMRI task. On each trial, subjects clicked a start button at the bottom center of the screen, which was followed by a face stimulus in the same location. Subjects were instructed to categorize the face’s gender (male or female) or emotion (angry or happy) as quickly and accurately as possible by clicking one of the two response options located at opposite top-corners of the screen. During this process, the mouse trajectory was continuously recorded, which was used to estimate its deviation toward the opposite response (serving as an index of category co-activation during perception). Subjects had to make their categorization within 2000 ms of initiating the trial. Subjects completed two blocks, one gender categorization and one emotion categorization. The order of the blocks was counterbalanced across subjects, as was the position of response options (left/right). Each face stimulus was presented twice: once in each block, resulting in 280 trials overall.

#### Stereotype content task.

To index subjects’ stereotypical associations (male = angry, female = happy), conceptual similarity of the four categories was assessed with a ratings task used in previous work examining biases in gender and emotion perception (Brooks *et al.*, 2018). In four separate randomized blocks for the four categories (angry, happy, male and female), subjects rated to what extent each of 30 descriptors including bodily feelings, thoughts, and actions was conceptually related to the category in question on a 7-point Likert scale (e.g. ‘on a scale of 1 = not at all to 7 = extremely, please rate how well the phrase *loud* stereotypically describes a happy person’). This resulted in a total of 120 trials. As we were interested in subjects’ learned stereotypical associations, not necessarily what they personally endorse, subjects were instructed to base their answers on what a typical American might indicate (to avoid issues of social desirability bias), as in previous work (Stolier and Freeman, 2016; Brooks *et al.*, 2018).

### Analytic approach

To allow for a comparison between behavioral and neuroimaging data, we used representational similarity analysis (RSA) (Kriegeskorte *et al.*, 2008). RSA involves the measurement of the similarity (e.g. correlation) of all pairwise combinations of conditions in one particular variable (e.g. mouse-tracking data), which is then compared with other patterns of similarity values from alternative modalities (e.g. neuroimaging data). In this way, RSA is a method used to measure the correspondence of representations across different modalities (Kriegeskorte *et al.*, 2008). For our present purposes, RSA allows us to assess the extent to which an individual’s representational space of social-conceptually shaped perceptions maps across behavioral and neural modalities. Specifically, we first demonstrated that subjects’ perceptions of faces (as measured by mouse-tracking) were biased by stereotypical associations. We then used RSA to test the correspondence between such stereotypically biased similarity in subjective perceptions with the similarity of faces’ multi-voxel response patterns.

Given our a priori interest in how stereotypical associations affects ventral-visual representations of faces, we first focused on multi-voxel response patterns across the entire anatomical ROI of the right VTC. We subsequently corroborated and extended the results using whole-brain searchlight analyses that identified these effects across the brain.

### Experimental design and statistical analyses

#### Stereotype strength.

To index each subject’s strength of stereotypical associations, we calculated the Pearson correlation between all pairs of the 30-length vectors of descriptor ratings for each social category in the stereotype content task. For example, to obtain each subject’s social-conceptual similarity between the categories anger and male, we calculated the Pearson correlation between their anger vector of 30 ratings and male vector of 30 ratings. We then subtracted stereotypically incongruent pairs from congruent pairs [angry male + happy female] − [angry female + happy male]. This calculation resulted in a score for each subject that represented the extent to which they held the belief that males tended to be angrier than women and that women tended to be happier than men. Scores could range from 2 (stereotypical bias: male = angry, female = happy) to 0 (lack of bias) to −2 (counterstereotypical bias: male = happy, female = angry).

#### Subjective dissimilarity matrix (DM).

We used mouse-tracking data to estimate the extent to which subjects’ stereotypical associations were reflected in their perception of faces. Trials were excluded if they exceeded the 2000 ms time deadline (2.83% of trials) or if the response was incorrect (3.70% of trials). We used standard mouse-tracking preprocessing procedures (Freeman and Ambady, 2010). All mouse trajectories were rescaled into a standard coordinate space with [0,0] at the start location and normalized into 100 time bins using linear interpolation to enable averaging of their full length across multiple trials. In order to obtain a by-trial index of category co-activation, we calculated the maximum perpendicular deviation (MD) of each trajectory toward the opposite response option. MD in two-choice mouse-tracking tasks is a long-used measure of the degree to which the alternate category was co-activated during the categorization process (Spivey and Dale, 2006; Freeman and Ambady, 2010; Stolier and Freeman, 2017; Freeman, 2018). For example, the degree to which subjects deviated towards the ‘Angry’ response while categorizing male faces can be understood as higher perceptual similarity between angry and male categories. By using these values in the subjective DM, the DM comes to reflect the stereotypical biases of subjects’ perceptions. The subjective DM can then be used to predict multi-voxel response patterns showing corresponding biases.

In order to create a subjective DM from the mouse-tracking data, we followed the procedure of previous work (Stolier and Freeman, 2016). For each of the four stimulus conditions (angry male, angry female, happy male, happy female), a bias in the subject’s hand trajectory to select the unselected response on the opposite side of the screen (e.g. spatial attraction towards ‘Angry’ when categorizing a happy male face) was calculated as the average MD relative to the maximum possible MD [MD/max(MD)]. The inverse effect [1−(MD/max(MD)] was treated as the bias toward the selected response (e.g. bias toward ‘happy’ when categorizing a happy male face). In this way, the distance of the trajectory toward the unselected versus selected response served as a proxy for the similarity of the stimulus condition to the four response categories across the mouse-tracking categorization tasks (emotion and gender categorization).

**Fig. 2.**
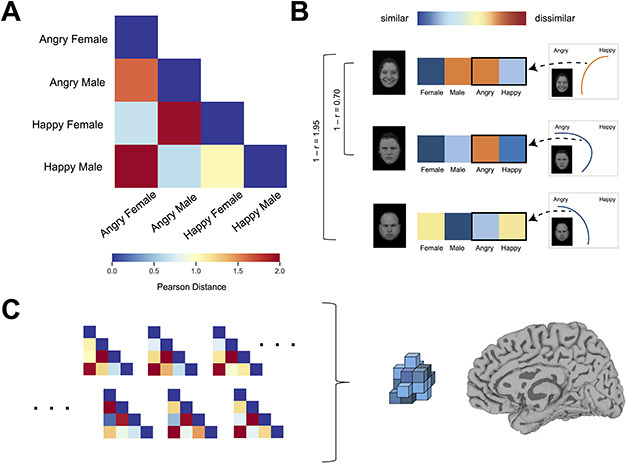
(A) The group-average subjective DM. Warmer colors represent greater dissimilarity. (B) The method by which a subject’s subjective DM was computed. In this example (Angry Male condition), MD values for the four categories (Female, Male, Angry and Happy), which reflect mouse trajectories’ attraction toward the four category responses in the mouse-tracking tasks, were used to create a response vector. Similarity in these response vectors was used to calculate pairwise similarity for the other conditions (Happy Female, Angry Female and Angry Male). In this way, face conditions that elicited similar activation of the Female, Male, Angry and Happy responses during mouse-tracking were deemed more similar in a subject’s subjective DM. (C) An illustration of our RSA procedure. Participants’ neural patterns for each condition were correlated with each other to formulate a neural DM. A subject’s own subjective DM was used to predict this neural DM (when controlling for the group-average subjective DM depicted in A).

Similarity measurements were calculated for each of the four conditions for all possible response options (emotion task: ‘angry’ and ‘happy’; gender task: ‘male’ or ‘female’). For each stimulus condition (angry male, angry female, happy male and happy female), this resulted in a four-length vector consisting of the similarity values toward each of the four category responses (angry, happy, male and female). We then computed the similarity between each of the respective four-length category-similarity vectors using the Pearson correlation distance (1−*r*) (Figure 2B), resulting in a 4 × 4 subjective DM for each subject (see Figure 2C for example subjective DMs). As such, the subjective DM for each subject captures the perceptual similarity between each pair of face conditions, in that the faces in those conditions activated the four response categories in a similar manner throughout the categorization tasks. In this way, the subjective DM reflects the extent to which social categories were biased toward one another in subjective perceptions. A group-average subjective DM was also calculated (Figure 2A) by taking the mean of all subjects’ similarity vectors and computing Pearson distances to produce dissimilarity measures between each averaged condition vector.

#### Stereotype congruency DM.

We computed a stereotype congruency DM reflecting our theoretical hypothesis that sex and emotion representations are biased according to their respective stereotypes. The DM was structured as follows: [1 (happy male), 2 (angry male); 2 (happy female), 1 (angry female)]. The cell values are Pearson distances and represent a stronger similarity between the pairs angry male and happy female compared to angry female and happy male.

#### Multi-level regression analyses.

Some behavioral analyses used multi-level regressions. There were conducted using generalized estimating equations (GEEs), which can incorporate nested data (repeated face stimuli nested within each subject) while accounting for the intracorrelations in repeated-measures designs (Liang and Zeger, 1986). We report unstandardized regression coefficients (*Bs*) and Wald *Z* values.

#### Discrimination task.

The post-scan gender discrimination task was analyzed using signal detection theory to control for response bias. We arbitrarily defined signal as male faces. Thus, the ability to discriminate male and female faces (*d*′) was computed as the proportion of masked male faces that were successfully categorized as male (hits), adjusted for the percentage of masked female faces that were erroneously categorized as male (false alarms): *d*′ = *z*-score (% hits)—*z*-score (% false alarms), with chance performance set at 0 ± 1.74.

#### fMRI acquisition.

Subjects were scanned using a Siemens 3T Magnetom Prisma with a 64-channel head coil at the New York University Center for Brain Imaging. Structural images were acquired using a 3D MPRAGE T1-weighted sequence with the following parameters: 2300 ms repetition time (TR); 2.32 ms echo time (TE); 0.9 mm^3^ voxel size; 230 mm field of view (FOV); 192 slices with no gap; anterior–posterior phase encoding direction. Functional images were acquired using a multiband echo-planar imaging sequence with the following parameters: 2000 ms TR; 35 ms TE; 2 mm^3^ voxel size; 208 mm FOV; 68 slices with no gap; anterior–posterior phase encoding direction and multiband acceleration factor of 4. Gradient spin-echo field maps were also acquired in both the anterior–posterior and posterior–anterior phase encoding directions for use in correcting for potential susceptibility artifacts. Diffusion-weighted images were collected at the end of the session, but those data are not reported here.

#### Data preprocessing and pattern estimation.

Image preprocessing was performed using FMRIPREP (Version 1.1.8) (Esteban  *et al.*, 2019), which is based on Nipype 1.1.3 (Gorgolewski *et al.*, 2011). The T1-weighted (T1w) image was corrected for intensity non-uniformity using N4BiasFieldCorrection (Tustison *et al.*, 2010) (ANTs 2.2.0) and used as T1w-reference throughout the workflow. The T1w-reference was then skull stripped using antsBrainExtraction.sh (ANTs 2.2.0), using OASIS as target template. Spatial normalization to the ICBM 152 Nonlinear Asymmetrical template version 2009c (Fonov *et al.*, 2009) was performed through nonlinear registration with antsRegistration (ANTs 2.2.0) (Avants *et al.*, 2008) using brain-extracted versions of both T1w volume and template. Brain tissue segmentation of cerebrospinal fluid, white matter and gray matter was performed on the brain-extracted T1w using fast (FSL 5.0.9) (Zhang *et al.*, 2001)

For each of the four BOLD runs per subject, the following preprocessing steps were performed. First, a reference volume and its skull-stripped version were generated using a custom methodology of *fMRIPrep*. Susceptibility distortions were corrected using 3dQwarp (Cox and Hyde, 1997) (AFNI). Based on the estimated susceptibility distortion, an unwarped BOLD reference was calculated for a more accurate co-registration with the anatomical reference. The BOLD reference was then co-registered to the T1w reference using flirt (FSL 5.0.9) (Jenkinson and Smith, 2001). Motion-correcting transformations, BOLD-to-T1w transformation and T1w-to-template (MNI) warps were concatenated and applied in a single step by using antsApplyTranforms in ANTs, using Lanczos interpolation.

We estimated the average hemodynamic response per voxel for each condition (using the 3dDeconvolve procedure in AFNI). BOLD responses were modeled by using a general linear model (GLM) with a design matrix that included a total of 12 predictors: 4 predictors for each stimulus condition and 8 predictors to model effects of no interest (average signal at each time point attributable to cerebrospinal fluid, white matter, global signal, linear motion in three directions and angular motion in three directions). Two separate GLM design matrices were constructed. One GLM modeled the four predictors of interest as angry female, happy female, angry male and happy male faces. For other analyses that required modeling trials as non-crossed categories, another GLM modeled the four predictors of interest as angry, happy, male and female. In both GLMs, all predictors of interest were modeled as boxcar functions across the duration of each event (2000 ms), during which the stimuli were presented. The boxcar functions were convolved with a gamma variate function (GAM in AFNI). The voxelwise *t* statistics associated with each of the four stimulus conditions were averaged across runs and the resulting maps were *z*-normalized and used as whole-brain patterns of activation for each face category for use with multi-voxel pattern analyses (MVPA).

#### Multi-voxel pattern analyses.

All MVPAs were performed using PyMVPA (Hanke *et al.*, 2009). Due to an a priori hypothesis regarding multi-voxel response patterns in the right VTC, we defined an anatomical region of interest (ROI) of the right VTC. The ROI mask was created using the Harvard–Oxford Cortical Structural Atlas in FSL and consisted of all atlas regions within the right VTC, totaling 7345 voxels. In the ROI analyses, the neural DM was constructed by calculating the Pearson correlation distance between all condition pairs using all voxels within the ROI (7345-length vectors). This resulted in a neural DM for each subject, representing the similarity of neural patterns relating to different social categories.

In whole-brain searchlight analyses, a searchlight sphere of 123 voxels (three voxel radius) was centered on a given voxel, which was iterated across all voxels in the brain. At each searchlight, a neural DM was constructed by calculating the Pearson correlation distance between all condition pairs for voxels within the sphere (123-length vectors). In all cases, DMs were vectorized and comparisons were made by the way of Spearman rank correlation so as to not assume a linear relationship. When a covariate DM needed to be statistically adjusted (e.g. subjective DM and neural DM, while controlling for group-average subjective DM), multiple regression RSA using rank-ordered pattern vectors was used. For ROI analyses, subjects’ Spearman rho values or regression beta values were submitted to a one-sample *t*-test against 0 (Spearman rho values were first Fisher-z transformed). For searchlight analyses, the resulting regression beta value was remapped back to the searchlight center voxel, yielding subject-level maps. These maps were then smoothed using AFNI’s 3dBlurToFWHM at 6-mm FWHM and tested at the group level by using a one-sample *t*-test together with maximum statistic permutation testing via Randomize in FSL (Winkler *et al.*, 2014), which tested significance of the raw *t* statistic over 5000 permutations. The subsequent group-level statistical maps were thresholded at the *P *< 0.05 level and corrected for multiple comparisons using threshold-free cluster enhancement (TFCE; Smith and Nichols, 2009).

#### Psychophysiological interaction analysis.

In order to support the theoretical assumption that the right VTC has diminished functional connectivity with frontal regions during masked relative to unmasked presentation of faces, we performed a psychophysiological interaction (PPI) analysis. To obtain our seed region, we conducted a whole-brain univariate contrast of unmasked > masked conditions to identify face-sensitive regions, i.e. those more responsive to processing unmasked faces than noise patterns. As this is a univariate analysis, maps were first smoothed using a Gaussian filter (6-mm FWHM) before submitting to the whole-brain contrast. To obtain an inclusive region of the right FG for use as a seed region (not for inferential purposes), we used a liberal voxelwise threshold of *P *< 0.05 (minimum cluster extent = 20 voxels). This analysis revealed an extensive portion of the right FG (see Results). We used this right FG cluster as a seed in our PPI analysis, identifying any neural regions that exhibited enhanced functional connectivity with the seed region during viewing of unmasked faces as compared to masked faces. We extracted the average BOLD series time course across all voxels within the seed region and deconvolved the time course with a gamma variate hemodynamic response function (GAM in AFNI). A GLM design matrix was constructed with three predictors: the seed time course, condition (masked = −1, unmasked = 1) and, most critically, the PPI interaction term. The resulting beta values for the PPI interaction term maps were then tested at the group level by using a one-sample *t*-test. Correction for multiple comparisons was performed using TFCE. The subsequent group-level statistical maps are, thus, significant at the *P *< 0.05 level and corrected for multiple comparisons.

## Results

### Behavioral results

Subjects’ stereotype scores indexed the strength of their stereotypical associations, with 2 reflecting strong stereotypical bias (male = angry, female = happy), 0 a lack of bias and −2 strong counterstereotypical bias (male = happy, female = angry) (see Materials and Methods). A one-sample *t*-test confirmed that overall subjects showed a significant stereotypical bias, associating men with anger and women with happiness (*M* = 0.49, SD = 0.62), *t*_(37)_ = 4.83, *P *< 0.0001.

To test whether these stereotypical associations were reflected in subjects’ perceptions of faces (assessed via mouse-tracking), we regressed maximum perpendicular deviation (MD) values onto face gender (male = −0.5, female = 0.5), face emotion (angry = −0.5, happy = 0.5), subjects’ stereotype scores (mean-centered) and their interactions using multi-level GEE regression. There was a significant main effect of emotion, *B* = −0.06, SE = 0.01, 95% CI [−0.08, −0.04], *Z* = −5.35, *P *< 0.0001, which was qualified by a significant gender *x* emotion interaction, *B* = −0.15, SE = 0.02, 95% CI [−0.19, −0.11], *Z* = −6.95, *P *< 0.0001. This interaction arose because mouse trajectories for stereotypically incongruent faces elicited a simultaneous attraction toward the opposite category response relative to stereotypically congruent faces. Specifically, trajectories for angry female faces (*M* = 0.50, SD = 0.54) were more partially attracted toward the opposite response than trajectories for angry male faces (*M* = 0.41, SD = 0.51), simple *B* = 0.091, SE = 0.02, 95% CI [0.06, −0.12], *Z* = 5.76, *P *< 0.0001; and trajectories for happy male faces (*M* = 0.43, SD = 0.52) were more partially attracted toward the opposite response than trajectories for happy female faces (*M* = 0.38, SD = 0.49), simple B = −0.06, SE = 0.01, 95% CI [−0.09, −0.03], Z = −4.00, *P* = < 0.0001. These stereotypic congruency effects were further qualified by a marginally significant three-way interaction, *B* = −0.057, SE = 0.035, 95% CI [−0.13, −0.01], *Z* = −1.68, *P* = 0.093. The gender *x* emotion interaction was exacerbated at higher levels (+1 SD) of stereotype strength (B = −0.1852, SE = 0.030, 95% CI [−0.24, −0.13], *Z* = −6.31, *P *< 0.0001) and attenuated at lower levels (−1 SD) of stereotype strength (*B* = −0.113, SE = 0.031, 95% CI [−0.17, −0.05], *Z* = −3.61, *P *= 0.0003). Thus, subjects with stronger stereotypical associations linking men to anger and women to happiness had a greater bias to perceive male faces as angry and female faces as happy.

As the three-way interaction effect has been previously found to be robust in larger samples (Brooks *et al.*, 2018), it is likely that here the three-way interaction result reached only marginal significance due to limited statistical power associated with the more moderate sample size necessary for neuroimaging. To confirm this, we repeated the behavioral tasks (mouse-tracking and stereotype content tasks) in a larger sample on Amazon Mechanical Turk (*N* = 142, 49 females, mean age = 34.5 years). We conducted an analogous GEE regression model for this direct replication, and we again observed strong stereotype congruency effects, i.e. a gender × emotion interaction, *B* = −0.115, SE = 0.0096, 95% CI [−0.13, −0.10], *Z* = −11.89, *P *< 0.0001. Moreover, in the direct replication, the three-way interaction was significant, *B* = −0.0361, SE = 0.0164, 95% CI [−0.07, −0.004], *Z* = −2.20, *P* = 0.028. As before, the three-way interaction arose because the gender *x* emotion interaction was exacerbated at higher levels (+1 SD) of stereotype strength (*B* = −0.1346, SE = 0.0129, 95% CI [−0.17, −0.11], *Z* = −10.43, *P *< 0.0001) and attenuated at lower levels (−1 SD) of stereotype strength (*B* = −0.0945, SE = 0.014, 95% CI [−0.12, −0.07], *Z* = −6.93, *P *< 0.0001). This replication provides additional support for the behavioral results of our fMRI sample and those of previous work (Brooks *et al.*, 2018).

### Neuroimaging results

One subject reported conscious awareness of the masked faces and was excluded from analysis. Another subject exhibited perceptual discriminability better than chance (*d*′* *± 1.74) in the post-scan discrimination task (*d*′* *=* *1.77) and was excluded; the remaining subjects’ *d*′ were low (*M* = 0.22, SE = 0.07), ensuring the masked faces were below these remaining subjects’ awareness.

Given our interest in how stereotypical associations are reflected in right VTC representations, we first conducted ROI analyses of the right VTC. A stereotype congruency DM was used to test for increased neural-pattern similarity for stereotypically congruent category pairs (angry male, happy female) relative to incongruent category pairs (angry female, happy male) using our hypothesized pattern of Pearson distances: [1 (happy male), 2 (angry male); 2 (happy female), 1 (angry female)]. Indeed, the structure of neural patterns in the right VTC was significantly correlated with the stereotype congruency DM in both the unmasked condition (mean rho = 0.30 one-sample *t*_(33)_ = 2.80, *P *= 0.008) and the masked condition (mean rho = 0.26; one-sample *t*_(33)_ = 2.60, *P* = 0.014). Moreover, the correlation of VTC pattern structure and the stereotype congruency DM did not differ between the unmasked and masked conditions, *t*_(33)_ = −0.21, *P *= 0.83.

These results demonstrate that the right VTC was indeed sensitive to both the unmasked and masked faces. However, they do not directly implicate the role of social-conceptual associations in neural-pattern structure as it is possible that physical properties of the face stimuli could still have accounted for this pattern. Although the conditions were equated on emotional intensity and low-level visual properties (luminance and contrast), the structural cues that convey anger and masculinity and that convey happiness and femininity do partially overlap (Becker *et al.*, 2007). As these cannot be fully equated (because faces could no longer be reliably categorized), linking effects to individual differences related to the strength of stereotypical associations provides more direct evidence for impact on perception when bottom-up and top-down overlaps are aligned (e.g. Brooks *et al.*, 2018). Thus, although the results thus far clearly show that the VTC was sensitive not only to the unmasked faces but also the masked faces, it is ambiguous whether these results reflect the effect of stereotypes or the effect of overlapping facial features. To test the role of stereotypes more specifically, we use individual difference analyses to link each subject’s stereotypically biased perceptions to neural response patterns.

A subjective DM was constructed for each subject using their pattern of stereotypically biased perceptual responses to faces via the mouse-tracking data (see Figure 2B). The mouse-tracking data thus far showed that subjects’ perceptions of male faces were biased toward anger and perceptions of female faces biased toward happiness, and that these effects were related to the strength of subjects’ stereotypic associations (male = angry, female = happy). We used multiple regression RSA to examine whether the neural-pattern structure in the right VTC reflected stereotypically biased subjective perceptions. Specifically, for each subject, we tested whether right VTC neural-pattern structure was significantly predicted by their subjective DM while controlling for the group-average subjective DM. Controlling for the group-average subjective DM is a conservative test that allows us to isolate the effect of subjects’ own unique biases in subjective perceptions and eliminate any common contributions shared across the sample or intrinsic to the stimuli. Multiple regression RSA confirmed that the right VTC neural-pattern structure conformed to subjects’ own subjective DM while controlling for the group-average DM in the unmasked condition (mean beta = 0.25; one-sample *t*_(33)_ = 2.57, *P *= 0.015). Critically, however, no such effect was observed in the masked condition (mean beta = −0.16; *t*_(33)_ = −1.74, *P* = 0.091), and in fact, there was a marginally significant trend in the opposite direction. (Note that the trend of a negative correlation is likely noise: negative RSA correlations are uninterpretable and researchers often preclude them altogether via non-negative least squares approaches; Khaligh-Razavi and Kriegeskorte, 2014). The correlation in the unmasked condition was also significantly stronger than that in the masked condition (*t*_(33)_ = 4.89, *P *< 0.0001). Thus, these results show that individual differences in stereotypically biased subjective perceptions are reflected in neural patterns of the right VTC while subjects viewed unmasked faces. When they viewed masked faces, however, VTC neural patterns ceased to reflect these stereotypically shaped individual differences. This was the case despite evidence that the right VTC clearly processed the masked face stimuli and was sensitive to their categorical distinctions.

**Fig. 3.**
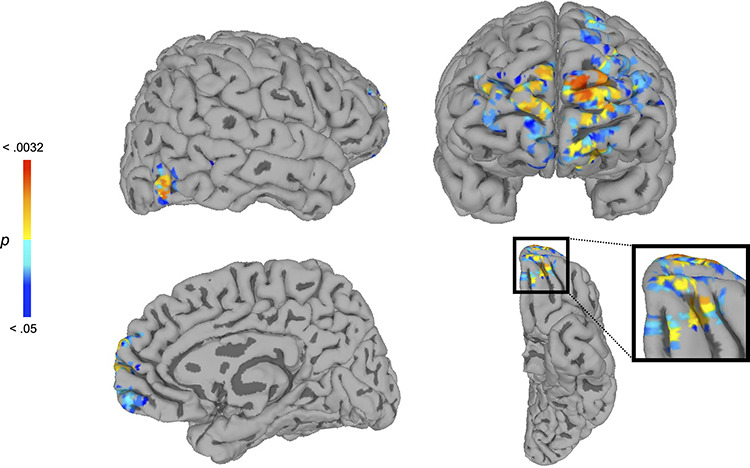
Whole-brain searchlight RSA results in the unmasked condition, revealing the right FG and mOFC. At each searchlight sphere, subjects’ own subjective DM was used to predict neural-pattern structure, while controlling for the group-average subjective DM. This analysis revealed regions in the right FG (top left), mOFC (bottom) and mPFC (top right), showing that these regions’ neural-pattern structure reflected subjects’ stereotypically biased subjective perceptions, even when any common bias shared across subjects or intrinsic to the stimuli are accounted for (*P *< 0.05, corrected). No regions survived correction for this analysis in the masked condition (*P *< 0.05, corrected).

To explore whether stereotypically shaped subjective perceptions were reflected in additional regions, we conducted whole-brain searchlight analyses to identify regions whose neural-pattern structure conformed to subjects’ own subjective DM while controlling for the group-average subjective DM. As shown in Figure 3, in the unmasked condition this analysis revealed clusters of the mOFC (*x* = −16, *y* = 58, *z* = −18, *P* = 0.0132, 224 voxels; *x* = 4, *y* = 58, *z* = −14, *P *= 0.0232, 101 voxels) and right FG (*x* = 49.1, *y* = −78.2, *z* = −6.45, *P *= 0.0084, 113 voxels), as well as the medial prefrontal cortex (mPFC) (*x* = −10, *y* = 70, *z* = 10, *P* = 0.0032, 940 voxels; *x* = 8, *y* = 72, *z* = 10, *P *= 0.0122, 557 voxels). No other regions survived correction (*P *< 0.05, corrected). In the masked condition, no clusters emerged that survived correction (*P *< 0.05, corrected). These results show that, while subjects viewed unmasked faces, neural-pattern structure in the right FG, mOFC and mPFC reflected subjects’ unique, individual differences in stereotypically biased subjective perceptions, but this ceased to be case while subjects viewed masked faces.

**Fig. 4.**
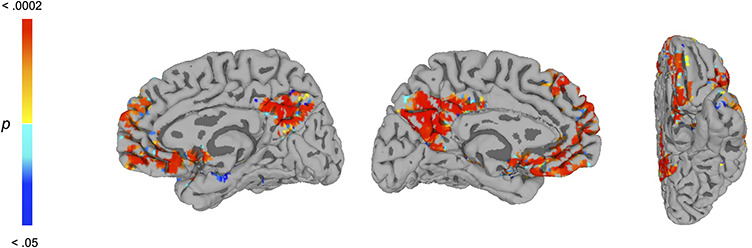
Whole-brain PPI analysis eliciting regions whose functional connectivity with the rFG seed region was modulated by masked *vs* unmasked conditions. A PPI analysis was conducted using a face-sensitive region of the right FG as a seed, revealing an extensive portion of the mOFC and additional regions involved in social cognition, including the mPFC (*P *< 0.05, corrected). These regions showed diminished functional connectivity with the right FG when subjects viewed masked as compared with unmasked faces. See Table 1 for list of regions.

To provide support for our theoretical assumption that feedback from frontal regions is reduced during masking, we tested whether patterns of functional connectivity with face-sensitive regions are diminished in the masked condition using a whole-brain PPI analysis. To obtain our seed region, we conducted a whole-brain univariate contrast of unmasked > masked conditions to identify face-sensitive regions (i.e. more responsive to unmasked faces than noise patterns) using a liberal threshold so as to define an inclusive region of the right FG for use as a seed (not for inferential purposes) (voxelwise *P *< 0.05, minimum cluster extent = 20 voxels). The contrast revealed sizeable portions of the right FG (*x* = 36, *y* = −54, *z* = −18, *t *= 7.58, 1230 voxels) and left FG (*x* = −38, *y* = −60, *z* = −18, *t* = 5.54, 711 voxels). We used the right FG region as the seed in our PPI analysis, seeking to identify any regions of the brain that showed diminished functional connectivity with this region during presentation of masked as compared with unmasked faces. Indeed, as hypothesized and shown in Figure 4, the PPI analysis revealed an extensive portion of the mOFC and, interestingly, a number of other regions involved in social cognition such as the temporoparietal junction and mPFC (see Table 1; *P *< 0.05, corrected). We also tested whether the PPI effect was observed in the specific mOFC regions elicited by the whole-brain searchlight RSA. For each subject, we extracted the PPI interaction term across the mOFC clusters from the searchlight RSA and submitted them to a one sample *t*-test against zero, which confirmed significant PPI effects (mean beta = 2.29; *t*_(33)_ = 3.72, *P *= 0.0007). These results show that functional connectivity between the mOFC (and other frontal regions) with the right FG was diminished when subjects viewed masked as compared with unmasked faces, consistent with previous work on backward masking’s disruption of frontal interactions with the ventral-visual pathway (e.g. Dehaene *et al.*, 2001).

**Table 1.** Regions elicited by the whole-brain PPI analysis (*P *< 0.05, corrected; coordinates denote peak voxel)

Region	Voxels	*x*	*y*	*z*	*P* value
Left inferior frontal gyrus	6691	−40	24	−18	0.0002
Left precuneus	1231	−6	−56	16	0.0002
Right precuneus	703	6	−54	16	0.0002
Left angular gyrus/temporoparietal junction	619	−52	−70	26	0.0004
Right middle temporal gyrus	521	60	2	−26	0.001
Left amygdala	430	−24	−6	−16	0.0006
Right parahippocampal gyrus	152	26	−24	−16	0.0074
Right angular gyrus/temporoparietal junction	75	46	−68	40	0.0086

We conducted a final analysis to test the hypothesis that the disruption in mOFC–FG functional connectivity caused by masking may predict the reduced representation of stereotypically biased perceptions in the FG. From the searchlight analysis, for each subject we extracted the beta for the subjective DM from the right FG cluster (using the model that controlled for the group-average subjective DM) for the unmasked and masked conditions, which was used to create a [unmasked beta−masked beta] difference score. These were then correlated with the extracted PPI interaction betas from the mOFC (see above). Indeed, we found that the reduced mOFC–FG functional connectivity due to masking (PPI interaction betas extracted from the mOFC) significantly predicted the extent to which masking reduced the FG’s representation of the subjective DM ([unmasked−masked] difference score), *r*_(32)_ = 0.331, *P* = 0.0279 (one-tailed test for directional hypothesis). This result empirically links masking’s disruption of mOFC–FG functional connectivity with the reduced representation of stereotypically biased perceptions in the FG.

## Discussion

Behavioral and neuroimaging studies have increasingly demonstrated that the perception of others’ faces is malleable to higher order social cognitive processes, including stereotypes, attitudes and goals. In the present research, we tested whether mOFC–FG cortical interactions are a mechanism by which stereotypes exert their influence on face perception. We found that the impact of one’s unique stereotypical associations on face-related representations in the right VTC including the right FG was strong when faces were normally presented. However, the stereotypical biasing of these representations was disrupted by masking, despite the fact that the VTC still retained the ability to process masked faces and was sensitive to their categorical distinctions. PPI analyses confirmed that the FG’s functional connectivity with the mOFC was disrupted despite spared processing of masked faces across the VTC. Moreover, the extent of masking’s disruption of mOFC–FG functional connectivity predicted a reduced representation of stereotypical associations in the FG. Critically, response patterns across the VTC and in the FG and mOFC exhibited a representational structure correlated with stereotypically biased subjective perceptions even when controlling for any common variance across the sample. Thus, these effects cannot be attributed to intrinsic physical features in the face stimuli themselves or common biases that were shared across the sample; instead, they reflect the impact of an individual’s unique, idiosyncratic social-conceptual knowledge on subjective perceptions. Taken together, our findings suggest that the effect of an individual’s social-conceptual knowledge on visual representations of faces does not arise from intrinsic processing within the VTC or FG alone but instead depends in part on top-down feedback from the mOFC during perception.

Our findings provide new evidence helping to resolve critical questions about the neural basis of stereotyping and the social perception of faces. While stereotypes (and other social-conceptual knowledge) have recently been shown to affect faces’ multi-voxel representations in the VTC and FG (Brosch *et al.*, 2013; Stolier and Freeman, 2016; Brooks *et al.*, 2019), it has remained unclear how persistent stereotypes’ ‘collateral damage’ is on these perceptual brain regions. For example, do these regions contain face representations that are stereotypically biased in a relatively permanent manner due to long-term learning, or are they shifted on-the-fly due to one’s stereotypical expectations? The current results not only provide a novel demonstration supporting the latter, more transient biasing, but they also directly implicate the mOFC in inducing these transient stereotypical biases. As such, the findings bolster models of social perception such as the DI model, which propose that mOFC–FG interplay plays a key role in the social-conceptual shaping of face perception (Freeman and Johnson, 2016).

Previous evidence in favor of top-down effects due to social-conceptual knowledge has used correlational techniques, such as predicting FG representational structure from conceptually imbued perceptions or conceptual ratings (Stolier and Freeman, 2016; Brooks *et al.*, 2019), or demonstrations that ventral-frontal regions (e.g. mOFC) take on some of the processing load of ventral-temporal regions when subjects have expectations about visual stimuli (Summerfield and Egner, 2009). Additionally, studies have shown that when subjects have expectations about face stimuli in particular, the mOFC exhibits enhanced top-down effective connectivity on the FG (Summerfield *et al.*, 2006; Summerfield and Egner, 2009). The current findings extend these prior results by providing evidence suggesting that the social-conceptual biasing of face-related FG representational structure is driven by functional interactions with the mOFC; when these functional interactions are disrupted, as with masking, the FG no longer shows evidence of social-conceptual impact.

Although our results are broadly consistent with predictive coding models that stress the importance of prior expectations during perception (Bar, 2004; Bar *et al.*, 2006; Summerfield and Egner, 2009; Otten *et al.*, 2017), other models attempting to capture the conceptual and semantic structure of the VTC including the FG have taken a theoretical perspective based on deep neural network architectures. From this perspective, VTC and FG representations do not require on-line feedback during perception but instead rely on both information contained within the stimuli and the supervision signal during learning to incorporate semantic information (Khaligh-Razavi and Kriegeskorte, 2014; Jozwik *et al.*, 2017). Accordingly, social-conceptual impacts should persist in VTC and FG response patterns even when functional connectivity with the mOFC is disrupted. Although the results are inconsistent with this perspective, they do not exclude the possibility that social-conceptual learning modifies intrinsic VTC and FG representations to some extent. For instance, the mOFC may provide a necessary signal for the VTC and FG to access social-conceptual knowledge but that knowledge, in theory, could still be embedded in local representations. The results clearly show, however, that any accurate and complete model of the conceptual shaping of VTC and FG representations ought to account for the role of functional interactions with the mOFC.

Our findings may also have implications for interventions seeking to reduce implicit social biases. Lab-based bias interventions have been found to successfully reduce individuals’ implicit biases, but these effects tend to dissipate after 3–4 days (Lai *et al.*, 2016). One possible reason for the sustaining effect of stereotypical associations may not only be that they are continually reinforced by one’s social environment (e.g. media representations), but also that perceptions of others’ faces are biased to be more consistent with one’s stereotypical expectations, which may reinforce those associations as a kind of ‘visual confirmation bias’. If stereotypical associations were to become intrinsically embedded in local VTC and FG representations through long-term learning, it is plausible that even after stereotypical associations were modified at a conceptual level (e.g. via a bias intervention or changes in the social environment) that they would persist in the VTC and FG. This might necessitate bottom-up visually based interventions to ‘recalibrate’ VTC and FG representations to be unbiased. However, by providing evidence that response patterns across the VTC and in the FG no longer reflect stereotypically biased perceptions once relatively isolated from the mOFC via masking, the results suggest that if stereotypical associations are modified at a conceptual level then the corresponding representational bias in the VTC and FG should follow suit. Future research could build on this work to better characterize mOFC–FG interactions in stereotypically biased perceptions, which could inform interventions aiming to reduce implicit social biases.

This research is not without its limitations. Although the use of backward masking as a method of reducing functional connectivity between the VTC and other cortical regions is well supported by previous work and our PPI analyses confirmed the successful disruption, it is still an indirect manipulation. Although overall an effective way to reduce frontal feedback to the VTC (Dehaene and Changeux, 2011; Baars *et al.*, 2013), the exact level at which it disrupts frontal feedback in any given subject cannot be controlled. Future studies could extend this work using manipulations of functional activity (e.g. TMS) and more fine-grained measures of temporal dynamics (e.g. MEG; see Kveraga *et al.*, 2007). Furthermore, while there is robust evidence showing that masked face stimuli are perceptually encoded across the VTC and in the FG (Jiang and He, 2006; Sterzer *et al.*, 2008; Brooks *et al.*, 2012), future work could extend our findings by directly manipulating the degree to which such encoding takes place. Nevertheless, using backward masking in tandem with multivariate fMRI and RSA may provide a promising approach to explore the nature of ventral-visual representations once relatively disconnected from their wider cortical interactions. Finally, given our perspective that mOFC–FG interplay is a mechanism by which any form of social-conceptual associations—whether gender, racial or age stereotypes; emotion or trait knowledge; or otherwise—exert their influence over face perception (Freeman *et al.*, 2020), further masking studies need to test the generalizability of the present results.

In summary, here we provided evidence that the impact of stereotypes on visual representations of faces in the VTC and FG does not persist indefinitely via long-term learning but instead is transiently induced via mOFC–FG interaction. When that interaction is disrupted, as with masking, VTC and FG representations no longer show evidence of stereotypical bias. Thus, these findings suggest that the effect of our learned social-conceptual associations on visual processing of faces does not arise from within the VTC and FG alone, but instead depends in part on interaction with the mOFC during perception.
